# Social acquisition context matters: Increased neural responses for native but not nonnative taboo words

**DOI:** 10.3758/s13415-021-00951-4

**Published:** 2021-11-01

**Authors:** Katherine Sendek, Grit Herzmann, Valeria Pfeifer, Vicky Tzuyin Lai

**Affiliations:** 1 Department of Psychology, Neuroscience Program, The College of Wooster, 1189 Beall Ave, Wooster, OH 44691 USA; 2 Department of Psychology, University of California, Davis, 1 Shields Avenue, Davis California, 95616 USA; 3 Department of Psychology & Cognitive Science Program, University of Arizona, 1503 E University Blvd, Tucson, AZ 85721 USA

**Keywords:** taboo words, lexical decision, ERP, emotion, dialects, social words

## Abstract

This study examined whether the context of acquisition of a word influences its visual recognition and subsequent processing. We utilized taboo words, whose meanings are typically acquired socially, to ensure that differences in processing were based on learned social taboo, rather than proficiency. American English-speaking participants made word/non-word decisions on American taboo (native dialect), British taboo (non-native dialect), positive, neutral, and pseudo- words while EEG was recorded. Taboo words were verified as taboo by both American and British English speakers in an independent norming survey. American taboo words showed a more positive amplitude of the Late Positive Complex (LPC), a neural correlate of emotionality and social processing, compared with British taboo words and all other word categories. Moreover, in an item-wise analysis, LPC amplitudes of American taboo words were positively correlated with their taboo ratings. British taboo words did not show this effect. This indicates that American participants, who had very limited social contact with British English, did not have the same perception of social threat from British taboo words as they had from American taboo words. These results point to the importance of social context of acquisition in establishing social-affective meaning in language.

## Introduction

Are words learned during social interaction perceived differently compared with words learned from textbooks in school? The *emotional context of learning hypothesis* (Caldwell-Harris 2014; Harris et al. 2006) suggests the environment in which a language is learned and used (e.g., naturalistic vs. academic) influences the level of emotional impact the language has on the speaker. Words gain their emotional meaning from being acquired in emotional contexts. Studies that examined the perceived emotionality of words in a first language (L1) and a second language (L2) found that there is indeed a stronger emotional connection to L1 (Dewaele 2010, 2011). In addition, these results are modulated by social interaction. For instance, individuals who have romantic partners that they communicate with in an L2 report that L2 is their most emotional language (Dewaele 2011). Alternative to the *emotional context of learning* view, others suggest that the age at which a language is acquired (Harris 2004) or the proficiency in that language (Eilola et al. 2007; Harris 2004) are the factors that influence affective perception in words. In the current study, we investigated the *emotional context of learning* hypothesis, by examining the neural correlates of American and British taboo words in American English speakers.

Taboo words offer a unique method to study social influence on word learning and test the *emotional context of learning* hypothesis. Similar to emotional words, taboo words may require learning and use in a social context to acquire their taboo meaning. Taboo words are highly arousing, negative, offensive, and socially inappropriate (Janschewitz 2008). They represent a social, rather than a physical, threat (Wabnitz, Mortens, & Neuner, 2012). In other words, they are both socially and emotionally significant (Donahoo and Lai 2020). Developmentally, taboo word meanings are learned through social interaction (Vingerhoets et al. 2013) and cultural understanding (Jay 2009). This “tabooness” is clear in L1, given that the speaker is raised in a rich social and emotional environment where the associated taboo is learned from a young age through social interaction, such as peer interactions. In L2, the social impact of taboo acquisition may be less clear, especially if language and taboo meaning are learned in an academic environment, with the student merely being told the taboo meaning of a word. To illustrate, taboo words whispered by children out of earshot of authority figures can involve thrill and risk of reprimand, which is a very different taboo experience compared to when words are simply labeled as offensive.

Dialects provide a unique opportunity to study social and affective perception of words. Throughout this study, taboo words from participant’s nonnative dialect (British taboo words) will be used as an analog for words learned in a nonnaturalistic context, such as L2 words acquired in an academic setting. Using nonnative taboo words eliminates proficiency confounds seen in previous studies, because some dialects are considered mutually intelligible classes of the same language (Voegelin and Harris 1951). This is supported by Martin et al. (2016) who demonstrated that British English speakers easily understood American English words, without showing an increase in the neural correlate that indexes effort of lexical retrieval (N400). Likewise, Dewaele (2015) showed similar results for American English speakers with regard to British English words using surveys. Thus, British English and American English in written form can be considered as mutually intelligible, at least in the context of the current experiment. We are not suggesting that all dialects are mutually intelligible. For instance, Bühler et al. (2017) investigated speech in Standard German and Swiss German and found an increase in the neural correlate that indexes detection of phoneme variant (mismatch-negativity or MMN) when participants made similarity judgements.

Proficiency has been shown to influence emotional factors, such as self-rated perceived offensiveness in L2 (Wang 2019). This may have influenced the results of previous studies comparing L1 and L2 perception for both emotional (Anooshian and Hertel 1994; Brase and Mani 2017; Conrad et al. 2011; Chen et al. 2015) and taboo words (Eilola and Havelka 2011; Harris et al. 2003; Harris 2004), wherein proficiency was not controlled for. Given such mutual intelligibility by these “bi-dialectals” (Dewaele 2015), the use of dialects that are largely similar to each other, such as American English and British English, may remove the confound of L2 proficiency. Additionally, the use of a lexical decision task (LDT), in which people decide whether a letter string is a word or not, can ensure that words from the nonnative dialect are understood as words by participants, while sensitivity to emotional processing is retained (Conrad et al. 2011; Opitz and Degner 2012; Chen et al. 2015).

Several behavioral studies have provided evidence for differences in emotional and taboo word perception in L1 compared with L2. In word recall data, Anooshian and Hertel (1994) found better spontaneous recall of emotional words in L1, but not L2. However, more recent attempts to replicate this have shown no difference in the recall rates of emotional words in L1 or L2 (Ferre et al. 2010). Such discrepancy may be due to task differences. Aycicegi-Dinn and Caldwell-Harris(2009) found an increase for recalling emotional words and phrases in L2 in association, translation, and counting tasks, while an L1 increase for recalling emotional words was only seen in emotion rating tasks. In addition, manipulating the emotional context of learning can influence later word recall. Brase and Mani (2017) taught rare words in both languages to German/English bilinguals using either a neutral or emotionally rich video context. There was greater recall for L1 words in the neutral context, but there was no difference in recall between languages when words were learned in rich context.

In reaction time (RT) data, studies of emotional and taboo words in L1 and L2 showed that valence and taboo influence latencies in both sets of languages, albeit more pronounced in L1. Studies using LDT typically showed that negative words are recognized more slowly than neutral and positive words in L1 (Kuperman et al. 2014). Studies have also shown that there is a language by valence interaction only in L1, wherein emotionally valenced words have higher accuracy (Conrad et al. 2011) or faster recognition (Chen et al. 2015; Ponari et al. 2015) as compared to neutral words. Emotional priming effects have been seen in both L1 and L2 (Kazanas and Altarriba 2016). In terms of taboo words, they have been shown to have slower reaction times than non-taboo words (Sulpizio et al. 2019) and negative words (Donahoo and Lai 2020), while their accuracies in LDT do not significantly differ from other words in L1. In taboo Stroop tasks, bilinguals showed taboo related delays in response time to both L1 and L2 (Eilola and Havelka 2011; Eilola et al. 2007). Lastly, in an attentional blink paradigm, taboo words delayed responses in both L1 and L2, but the delay was greater in L1 than in L2 (Colbeck and Bowers 2012). Given these results, taboo status of words is maintained in L2, though its influence may differ from or be reduced in L2 as compared to L1.

Differences in emotional and taboo salience of L1 and L2 have also been observed in physiological data. Based on skin conductance response, bilinguals experience increased arousal in L1, but not L2, when making emotional or morality-based choices in decision making tasks (Lazar et al. 2014). Similarly, taboo words elicit increased arousal compared with meaning matched euphemisms (Bowers and Pleydell-Pearce^2011^). In emotional language, the directionality of effects is inconsistent across studies. Some reported that bilinguals showed increased arousal to certain types of words (swears, reprimands) in their L1 (Eilola and Havelka 2011; Harris et al. 2003), whereas other studies showed these increases in L2 (Aycicegi-Dinn and Caldwell-Harris^2009^). However, the increases in skin conductance seen in L2 may be influenced by age and context of acquisition (Harris 2004) as well as language context of the experiment (Aycicegi-Dinn and Caldwell-Harris^2009^). For instance, simultaneous, early bilinguals showed no difference between L1 and L2 (Harris 2004). These physiological results were corroborated by survey data and anecdotal accounts from proficient bilinguals, who reported L1 being more emotional and the language of choice for taboo word use (Dewaele 2010, 2011). However, taboo words were reported as being more cathartic when L2 was learned in a naturalistic context (Dewaele 2017).

Event related potentials (ERPs) are sensitive to both language and emotion processing in L1 and L2. The N400 component is a negative potential, peaking between 300-500 ms, that reflects lexical retrieval. In an LDT, pseudowords typically elicit a more negative N400 amplitude than meaningful words (Kutas and Federmeier 2011). The N400 amplitude can be influenced by familiarity, for example dialect-level sound and semantic familiarity, however this is limited to auditory presentation (Bühler et al., 2017; see Martin et al. 2016 for contrasting results in auditory presentation). Differences in emotional processing between L1 and L2 are seen later, particularly in the late positive component (LPC). The LPC is a positive potential, peaking between 500-800 ms (Citron 2012). According to Citron (2012), the LPC reported in emotional language studies is an explicit indicator of emotionality, requiring directed reprocessing of the target word. It is typically enhanced for emotional words in L1 (Citron 2012). Studies have found a less positive LPC amplitude for emotional words in L2 compared to L1 (Chen et al. 2015), as well as other LPC effects (Opitz and Degner 2012). These data support a tendency for greater perceived emotionality of valenced words in L1 compared with L2, which is congruent with previously described qualitative reports of language use by bilinguals (Dewaele 2011).

Only a few ERP studies have examined taboo words. In Wabnitz et al. (2012), passive reading of emotional and taboo words elicited increased P100 for taboo, but not for valence and arousal matched positive or negative words. In Donahoo and Lai (2020), a taboo effect was seen in the LPC using a LDT paradigm. Despite the difference in time-course, these results indicate that taboo words are processed differently than matched emotional words. In individuals with social anxiety disorder and posttraumatic stress disorder, reading taboo words passively led to an increase in a late (500-800 ms) alpha activity (Wabnitz et al. 2016) and a more positive LPC amplitude (Klein et al. 2019), respectively. These findings likely reflect hypersensitivity to social cues in these two clinical groups. The ERP studies of taboo words informed us that taboo words are perceived as distinct from emotional words, being socially threatening, and that their processing is often reflected in an increase in late ERPs, particularly the LPC.

The present study investigated how context of acquisition influences affective word perception, where emotional context of learning was manipulated via taboo words and dialects. Native speakers of American English, who had little direct or indirect interaction with British speakers and British English, performed a LDT on American English and British English taboo words, negative words whose valence was matched with taboo words, positive words, neutral words, and pseudowords. The words used in the experiment were assessed for comprehension, both in a norming survey beforehand and by participants after the experiment, to ensure that participants, despite not having had social contact with British English, still understood the words used in the study. Positive words were included to capture the full spectrum of valence, as well as emulate previous studies (Chen et al. 2015; Conrad et al. 2011; Opitz and Degner 2012; Sulpizio et al. 2019). If the social nature of taboo words (Donahoo and Lai 2020; Janschewitz 2008; Reilly et al. 2020) depends on their acquisition in naturalistic, social interactions, then taboo words in the native dialect were expected to show a heightened affective perception, in the form of a more positive LPC amplitude, while those in the non-native dialect were not. However, if tabooness depends on proficiency, which refers to the general English proficiency, regardless of British or American dialect, of our American English speakers in this specific experiment context, no difference was expected between native and non-native taboo words. Based on results by Donahoo and Lai (2020), we also expected more positive LPC amplitude for native taboo words (American), as compared to all categories of non-taboo words (negative, positive, neutral). For nonnative taboo words (British), on the other hand, we expected no difference in LPC compared with native, nontaboo words, given their lack of social context of acquisition. Finally, for verification of the experimental manipulation, pseudowords were predicted to show more negative N400 amplitudes compared with words (Kutas and Federmeier 2011).

## Methods

### Participants

Participants were 23 undergraduates (mean age = 19, *SD* = 0.91, handedness 21R/2L) recruited from an Introduction to Psychology course. Sixteen participants identified as female, seven as male, none reported intersex, and none preferred not to answer. All participants had normal or corrected-to-normal vision. Participants gave written, informed consent and received course credit for their participation. This study was approved by The College of Wooster Human Subjects Review Committee. Using G*Power (version 3.1.9.4 Faul et al. 2007) and data from Opitz and Degner (2012), it was determined that, in order to replicate previous findings relating to emotional words in L1 and L2, a sample of 20 participants would be required to reach a power level of 0.95 (*alpha* = 0.05). Given the differences between the current design and previous studies, as well as the exploratory nature of the current study, the minimum number of participants was slightly increased.

All participants had American English as their L1, lived in the United States for the majority of their lives, had minimal direct interaction with speakers of British English, and used American English taboo words regularly, as determined by an extensive language background questionnaire (adapted from *The Language Contact Profile*, Freed et al. 2004; Appendix 16). Language background data are available in Appendix B.

### Stimuli

The stimuli consisted of 120 neutral, 30 negative, 30 positive, 30 American English taboo, 30 British English taboo, and 240 pseudo-words (Appendix C). These words were initially selected based on the Affective Norms for English Words (ANEW; Bradley and Lang 1999) and the English Lexical Project (ELP; Balota et al. 2007), and then were normed for tabooness and comprehension ratings, as well as valence and arousal ratings. All word properties for all word categories are reported in Table 1. The valence and arousal ratings replicated ANEW numbers. The word categories included in the EEG experiments were matched in terms of mean length, frequency, number of orthographic and phonological neighbors, and number of syllables, using the ELP. The pseudowords were matched for mean length and number of orthographic neighbors, also using the ELP.

**Table 1:** Word properties of stimuli based on Norming Survey, ANEW, and ELP

		American taboo	British taboo	Negative	Positive	Neutral
Data retrieved from ELP	Length	5.60 (1.89)	5.57 (1.74)	5.10 (1.35)	5.17 (1.09)	5.33 (1.20)
	Log Frequency	6.23 (0.99)	6.18 (1.34)	6.79 (0.89)	6.46 (0.93)	6.64 (0.97)
	Syllables	1.52 (0.57)	1.63 (0.56)	1.40 (0.56)	1.60 (0.62)	1.55 (0.59)
Data collected from American speakers (matched to ANEW)	Valence	3.65 (1.21)	3.79 (1.00)	3.89 (0.22)	6.80 (0.50)	5.17 (0.41)
	Arousal	4.97 (0.78)	4.61 (0.88)	4.97 (0.41)	5.01 (0.25)	4.82 (0.47)
	Tabooness	5.91 (1.34)	4.20 (1.13)	1.78 (0.38)	1.59 (0.39)	1.68 (0.54)
	Comprehension	4.51 (0.30)	3.65 (0.66)	4.35 (0.33)	4.45 (0.29)	4.39 (0.42)
Data collected from British speakers (matched to ANEW)	Valence	3.72 (1.12)	3.20 (0.87)	3.89 (0.22)	6.80 (0.50)	5.17 (0.41)
	Arousal	3.03 (0.79)	2.73 (0.85)	2.52 (0.34)	3.48 (0.75)	2.63 (0.42)
	Tabooness	4.87 (1.39)	4.40 (1.20)	2.09 (0.59)	1.65 (0.65)	1.72 (0.47)
	Comprehension	4.52 (0.58)	4.50 (0.39)	4.21 (0.47)	4.50 (0.25)	4.30 (0.43)

Norming was conducted to verify tabooness, comprehension, valence, and arousal. Two groups of participants were included: an American group and a British group. The American group was sampled from 104 MTurk workers (see Munro et al., 2010 for use of MTurk in language studies) who gave written informed consent. Eight were excluded due to English not being their L1, 14 were excluded due to incorrect attention check questions, and 20 were excluded due to their geographic location within a non-American English speaking country (19 India, 1 Brazil). The remaining 62 American participants had a mean age of 37 and were located in either the United States (61) or Canada (1). The British group was sampled from 87 participants who were recruited from Prolific (a UK-based site comparable to MTurk) who gave written, informed consent. Five were excluded due to a failure to complete the survey, and ten were excluded due to incorrect attention check questions. The remaining 63 British participants had a mean age of 38 and were located in England.

Stimuli in the Norming Survey included 47 negative, 42 positive, and 148 neutral words taken from the Affective Norms for English Words databases (ANEW; Bradley and Lang 1999) and normed for lexical characteristics and arousal using the ELP and ANEW databases. Thirty-nine American taboo (AT) words and 38 British taboo (BT) words were collected from Janschewitz (2008), Dewaele (2015), and native speakers of either dialect. Their lexical characteristics were checked and matched using the ELP.

Participants in the Norming Survey were asked to rate each word on a 9-point scale for tabooness (unoffended-offended), valence (negative-positive), and arousal (unexcited-excited). Instructions for the question on tabooness were taken from Janschewitz (2008): “How offensive the word is to people in general?” Participants were also asked to rate each word for “comprehension”—how well they understood the word on a 5-point scale (1 = little to no understanding” and 5 = “completely understand”). Words taken from the ANEW were only rated for comprehension and tabooness, because their valence and arousal ratings were available.

The norming results for the words included in the EEG experiment are summarized in Table 1 and the statistical results are summarized in Table 2. Overall, the valence, arousal, and tabooness ratings from American and British English speakers are mostly comparable and the group difference in comprehension ratings is minimum, which we will come back to in general discussion. We first examined if native speakers of British English and American English perceived the word categories differently. In terms of tabooness, AT words were rated significantly more taboo than negative, positive, and neutral words by American and British participants, respectively (AT vs. negative: *t*(58) = 16.24, *p* < 0.001, *d* = 4.19; *t*(58) = 12.01, *p* < 0.001, *d* = 2.09; AT vs. positive, *t*(57) = 15.23, *p* < 0.001, *d* = 4.38; *t*(57) = 11.10, *p* < 0.001, *d* = 2.97; AT vs. neutral, *t*(151) = 27.50, *p* < 0.001, *d* = 4.14; *t*(151) = 21.89, *p* < 0.001, *d* = 3.04)). BT words were also rated significantly more taboo than negative, positive, and neutral words by American and British participants, respectively (BT vs. negative, *t*(57) = 11.95, *p* < 0.001, *d* = 2.87; *t*(57) = 11.21, *p* < 0.001, *d* = 2.44; BT vs. positive, *t*(56) = 10.37, *p* < 0.001, *d* = 3.09; *t*(56) = 10.13, *p* < 0.001. *d* = 2.85; BT vs. neutral, *t*(150) = 17.94, *p* < 0.001, *d* = 2.85; *t*(150) = 19.73, *p* < 0.001, *d* = 2.94). AT words were significantly more taboo than BT for American participants, *t*(57) = 5.29, *p* < 0.001=, *d* = 1.38. There was no significant difference in tabooness between AT and BT for British participants, *t*(57) = 1.65, *p* = 0.10, *d* = 0.36. In arousal ratings, British participants rated words as being significantly less arousing in all categories, as compared to American participants, *t*(38) = 12.58, *p* < 0.001, *d* = 2.00, and ANEW, *t*(38) = 13.39, *p* < 0.001, *d* = 3.10, however, the arousal rating did not differ significantly between categories for the British, *F*(4,47) = 1.77, *p* = 0.15, *η*^*2*^ = 0.13, and the American participants, *F*(4,234) = 2.16, *p* = 0.075, *η*^*2*^ = 0.04. In terms of valence ratings, word valence did not differ significantly between American and British participants, *t*(38) = 1.13, *p* = 0.27, *d* = 0.08. In comprehension ratings, Americans rated their comprehension for BT words as significantly lower than all other categories, *F*(4,236) = 17.01, *p* < 0.001, *η*^*2*^ = 0.22 (Table 2), while there was no difference in comprehension for British participants, *F*(4,236) = 1.96, *p* = 0.10, *η*^*2*^ = 0.03.

**Table 2:** Independent sample *t*-values for American Norming Survey Comparisons

		Valence	Taboo	Comprehension
		*t*-value (df)	*t*-value (df)	*t*-value (df)
**American Taboo**				
	British Taboo	−0.49 (57)	5.29 (57)***	6.47 (57)***
	Negative	−1.10 (57)	16.23 (58)***	1.89 (58)
	Positive	−13.05 (57)***	16.92 (58)***	0.75 (58)
	Neutral	−11.46 (147)***	26.87 (145)***	1.49 (145)
**British Taboo**				
	Negative	−0.56 (58)	11.10 (57)***	−5.18 (57)***
	Positive	−14.92 (58)***	11.90 (57)***	−5.99 (57)***
	Neutral	−11.78 (148)***	17.50 (144)***	−7.43 (144)***
**Negative**				
	Positive	−28.88(58)***	1.88 (58)	−1.51 (58)
	Neutral	−16.52(148)***	1.00 (145)	−0.39 (145)
**Positive**				
	Neutral	18.31(148)***	−0.82 (145)	0.77 (145)

*** Significant differences (alpha < 0.001) and d > 1.34

In selecting words for the EEG experiment, based on the norming, 74 words were excluded (9 AT, 8 BT, 17 negative, 12 positive, and 28 neutral) so that length, *F*(5,474) = 0.59, *p* = 0.71, *η*^*2*^ = 0.01, log frequency, *F*(4,220) = 1.65, *p* = 0.60, *η*^*2*^ = 0.03, number of orthographic neighbors, *F*(5,459) = 1.38, *p* = 0.23, *η*^*2*^ = 0.02, number of phonological neighbors, *F*(4,220) = 0.42, *p* = 0.79, *η*^*2*^ = 0.01, number of syllables, *F*(4,234) = 0.71, *p* = 0.58, *η*^*2*^ = 0.01, and arousal, *F*(4,234) = 0.2.16, *p* = 0.075, *η*^*2*^ = 0.04, did not differ significantly between conditions (values for length and orthographic neighbors included pseudowords) with the stimuli that remained. Valence was matched between taboo and negative words. Verifying our design, positive and neutral words were rated significantly higher in valence and differed significantly from each other. Also verifying our design, positive, negative, and neutral words did not differ significantly in tabooness.

### Procedure

Participants filled out the language questionnaire and were then setup for EEG recording in a sound attenuated booth. During the experiment, they sat about 70 cm (visual angle maximally (i.e., for the longest word) 1.5° horizontally and consistently 0.5° vertically) away from the monitor. Stimuli were presented in black font (Courier New, size 18, bold) on a white background via E-prime 2.0 software (Psychology Software Tools, Inc.).

The task was a lexical decision task, where participants had to decide if a string of letters was a meaningful word in the English language or a nonword by pressing the 2 or 9 key on a keyboard with their left or right index finger. They were asked to work as quickly as possible while maintaining accuracy. Response key to word/nonword assignment was counterbalanced with subject number. The experiment consisted of 6 blocks with 80 trials in each block. Self-determined breaks were taken between blocks.

**Fig. 1:**
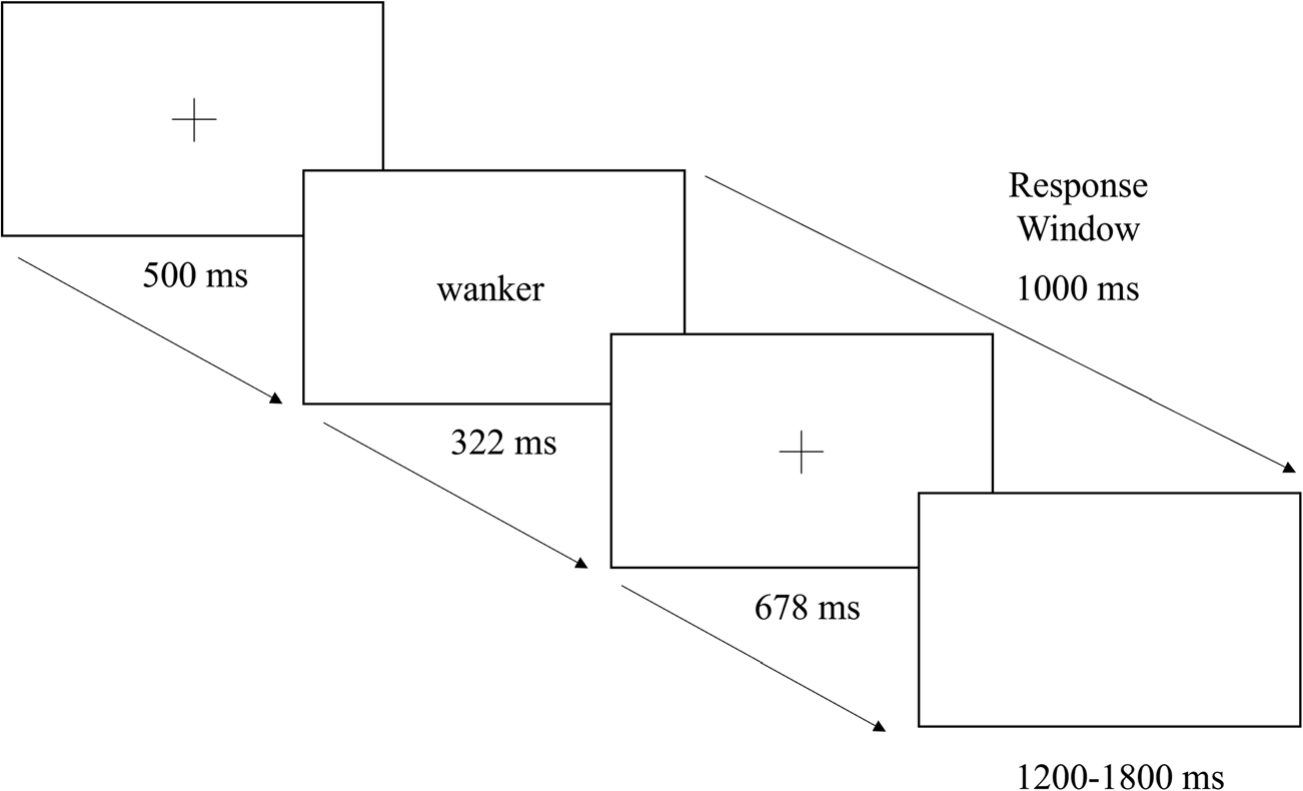
Example of a British taboo word trial

In each trial (Figure 1), participants saw a fixation cross for 500 ms, followed by the target word. The target remained on the screen for a period of time determined by the length of the word (100 ms + (37 ms * number of letters) for words less than 8 letters; 400 ms for words with more than 8 letters). The word was then replaced by a fixation cross, so that the total combined time for the presentation of the word and second fixation cross was 1,000 ms. Participants were asked to respond within the 1,000-ms time window after word onset. The intertrial interval was jittered between 1,200 ms and 1,800 ms.

After the EEG experiment, participants took a survey where they rated each of the taboo words (30 AT, 30 BT) for “comprehension”—how well they understood the word on a 5-point scale (1 = little to no understanding” and 5 = “completely understand”), just like the Norming Survey. Words in the EEG experiment and the post-EEG comprehension rating were presented in different random orders. The total experimental session lasted for about 75 minutes.

### ERP recording and measurement

The EEG was recorded continuously with active electrodes mounted in an electrode cap (Easy-CapTM) at the scalp positions Fz, Cz, Pz, Oz, FP1, FP2, F3, F4, F7, F8, FT9, FT10, FC1, FC2, FC5, FC6, C3, C4, CP1, CP2, CP 5, CP6, T7, T8, P3, P4, P7, P8, O1, O2, TP9, and TP10. Initial common reference was FP1. AFz served as ground. Impedances were kept below 15 kΩ. All signals were recorded with a band-pass filter of 0.01 to 100 Hz and a sampling rate of 500 Hz.

The data was processed using Brain Vision Analyzer 2.0.4 (2013). Data was segmented from 200 ms before stimulus onset to 1,000 ms after and re-referenced to the average of left and right mastoid. Data was low-pass filtered at 30-Hz with 60-Hz notch and aligned to a 200-ms baseline before target onset. Ocular movements were corrected with an Independent Component Analysis algorithm (Infomax), and trials that included nonocular artifacts were removed automatically using the following criteria: voltage steps greater than 70 μV/ms, differences in values greater than 200 μV in a 200 ms segment, data points lower than −200 μV, and data points greater than 200 μV. No participants were removed due to artifacts. EEG data was averaged for each channel and condition. Participants included in the statistical analyses had a minimum of 15 trials per condition. The averages and standard deviations for trial counts in each condition were: AT (*M* = 26, *SD* = 2.6), BT (*M* = 21, *SD* = 4.0), negative (*M* = 25, *SD* = 3.8), positive (*M* = 27, *SD* = 3.0), neutral (*M* = 100, *SD* = 11), and pseudoword (*M* = 180, *SD* = 29).

Time windows and electrode locations were chosen based on literature (Chen et al. 2015; Citron 2012; Donahoo and Lai 2020; Kutas and Federmeier 2011) and visual inspection of the data (Figures 4 and 5). The N400 (300-500 ms) and LPC (500-700 ms) were analyzed along an extended midline Fz, Cz, Pz, FC1, FC2, CP1, and CP2.

### Statistical analysis

Participants with an average accuracy or reaction time (RT) more than 2 SD above or below the mean or less than 55% accuracy (approaching chance) for each category of words were excluded. Accuracy for words was determined using the number of accurate responses (hits) divided by the number of total responses given within the 1,000-ms response window in each condition. Accuracy for pseudowords was determined the same way but relied on the number of correct rejections rather than hits. Responses given after the 1,000-ms response window were excluded and were not counted as errors. Based on these criteria, 7 participants were excluded from the behavioral and EEG analysis. The final dataset contained 23 participants.

Accuracy and RT (correct responses only) were analyzed using repeated measures ANOVAs (*alpha* = 0.05) to determine the differences for each type of word (American taboo, British taboo, negative, positive, neutral, and pseudo-words). Comprehension ratings of AT and BT at the end the ERP experiment were analyzed with a paired-samples*t*-test.

For the N400, planned comparisons were conducted for pseudowords relative to each word category. Planned comparisons were used instead of an omnibus ANOVA, because the N400 was not central to the experimental question but instead used to check that the experimental manipulation (word/nonword) was successful.

For the LPC, a repeated measures ANOVA of 6 word type (American taboo, British taboo, negative, positive, neutral, and pseudowords) x 7 location (Fz, Cz, Pz, FC1, FC2, CP1, and CP2) was conducted (see Chen et al. 2015; Citron 2012; Donahoo and Lai 2020; Kutas and Federmeier 2011). Huynh-Feldt(1976) correction was used to account for violating the assumption of Sphericity. Uncorrected degrees of freedom are reported. All post-tests conducted from significant main effects or interactions were Bonferroni corrected for multiple comparisons.

## Results

### Behavioral Data

**Fig. 2:**
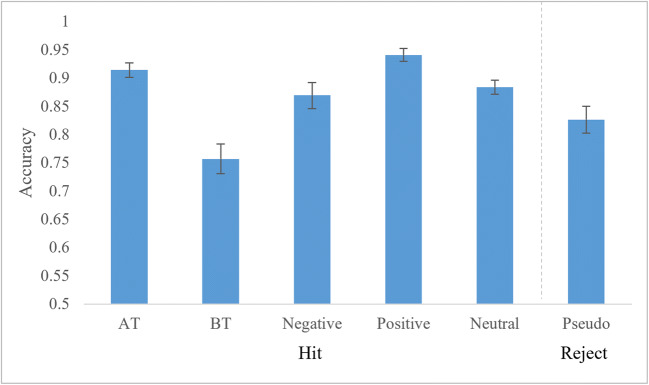
Lexical decision accuracy. Participants performed LDT for five categories of words and pseudowords (x-axis). Mean accuracies are shown on the y-axis (# of correct responses divided by # of correct responses and # of incorrect responses). Correct responses to pseudowords were categorized as rejections. Error bars indicate standard error

The accuracy results are summarized in Table 3 and Figure 2. Accuracy for words was determined using the number of accurate responses (hits) divided by the number of total responses given within the 1,000 ms response window in each condition. The repeated measures ANOVA yielded a main effect of word type, *F*(5,110) = 15.39, *p* < 0.001, *η*_*p*_^*2*^ = 0.412. BT had a significantly lower proportion of hits to incorrect rejections compared to all other categories of words: BT vs. AT: *t*(22) = 8.11, *p* < 0.001, *d* = 1.47, BT vs. negative: *t*(22) = 4.12, *p* < 0.001, *d* = 0.91, BT vs. positive: *t*(22) = 6.47, *p* < 0.001, *d* = 1.80, and BT vs. neutral: *t*(22) = 5.38, *p* < 0.001, *d* = 1.19. AT had a significantly higher proportion of hits than negative, *t*(22) = 2.17, *p* = 0.041, *d* = 0.45, and neutral words, *t*(22) = 2.25, *p* = 0.035, *d* = 0.49. Negative had a significantly lower proportion of hits than positive words, *t*(22) = 3.58, *p* = 0.002, *d* = 0.80. Neutral had a significantly lower proportion of hits than positive words, *t*(22) = 4.43, *p* < 0.001, *d* = 1.04. No other categories differed significantly. Comparison to pseudowords can be found in Appendix D.

**Table 3:** Behavioral results of LDT

	AT	BT	Negative	Positive	Neutral	Pseudo
Accuracy	0.91 (0.06)	0.76 (0.13)	0.87 (0.11)	0.94 (0.06)	0.88 (0.06)	0.83 (0.11)
RT (ms)	636 (62)	662 (70)	641 (68)	618 (62)	632 (63)	692 (61)

**Fig. 3:**
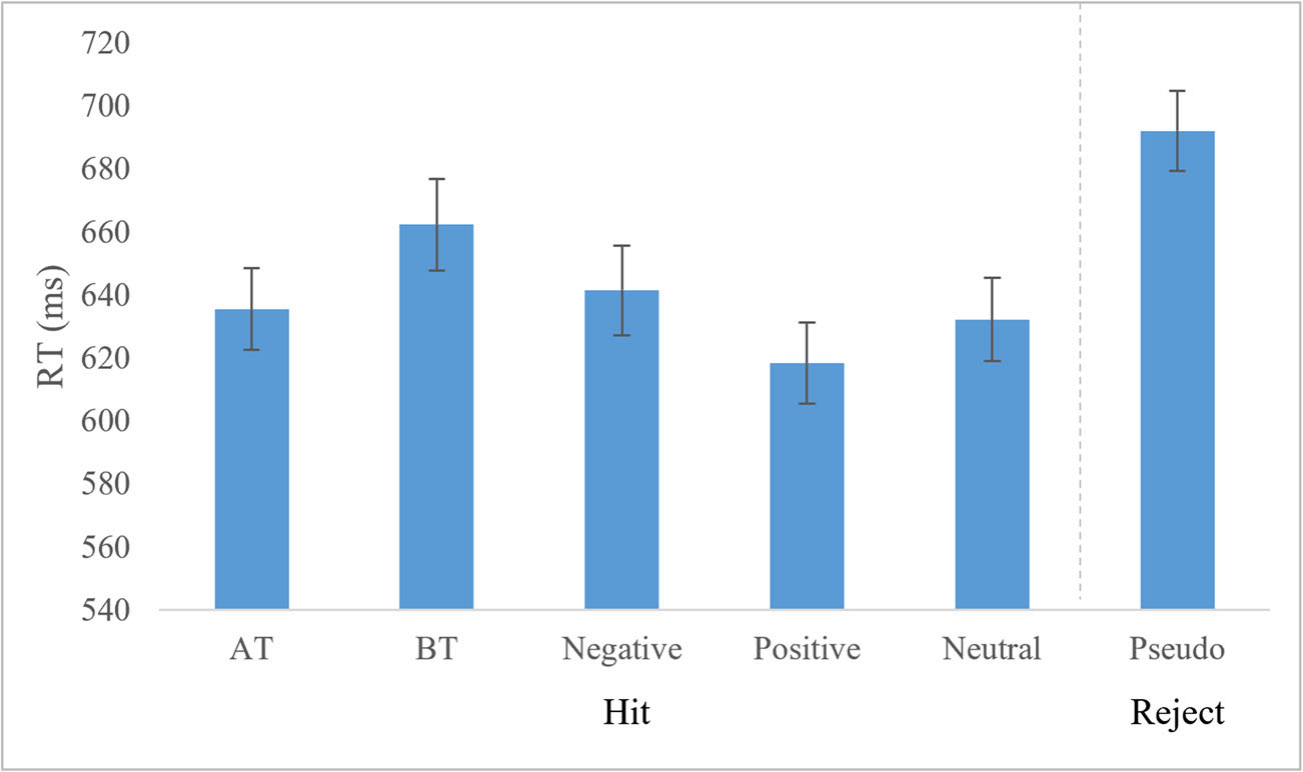
Lexical decision reaction times. RT for LDT for five categories of words and pseudowords were recorded (x-axis). Mean RTs for correct responses are displayed on the y-axis. Correct responses to pseudowords were categorized as rejections. Error bars indicate standard error

The RT results are summarized in Table 3 and Figure 3. The repeated measures ANOVA yielded a main effect of word type, *F*(5,110) = 21.17, *p* < 0.001, *η*_*p*_^*2*^ = 0.490. BT had significantly higher RTs than AT, *t*(22) = 3.01, *p* = 0.006, *d* = 0.39, positive, t(22) = 5.03, *p* < 0.001, *d* = 0.67, and neutral words, *t*(22) = 3.64, *p* = 0.001, *d* = 0.45. BT did not differ from negative words. Positive words had significantly lower RTs compared to all other categories; AT, *t*(22) = 2.64, *p* = 0.015, *d* = 0.29, negative, *t*(22) = 3.28, *p* = 0.003, *d* = 0.35, and neutral, *t*(22) = 2.93, *p* = 0.008, *d* = 0.22. No other categories differed significantly. Comparison to pseudowords can be found in Appendix D.

#### Post-EEG Comprehension Survey

A paired samples *t*-test of the comprehension ratings showed that AT (*M* = 4.55, *SD* = 0.37) had higher comprehension ratings than BT (*M* = 3.36, *SD* = 0.66), *t*(23) = 10.76, *p* < 0.001, *d* = 1.66.

### ERP Data

#### N400 (300-500 ms)

**Fig. 4:**
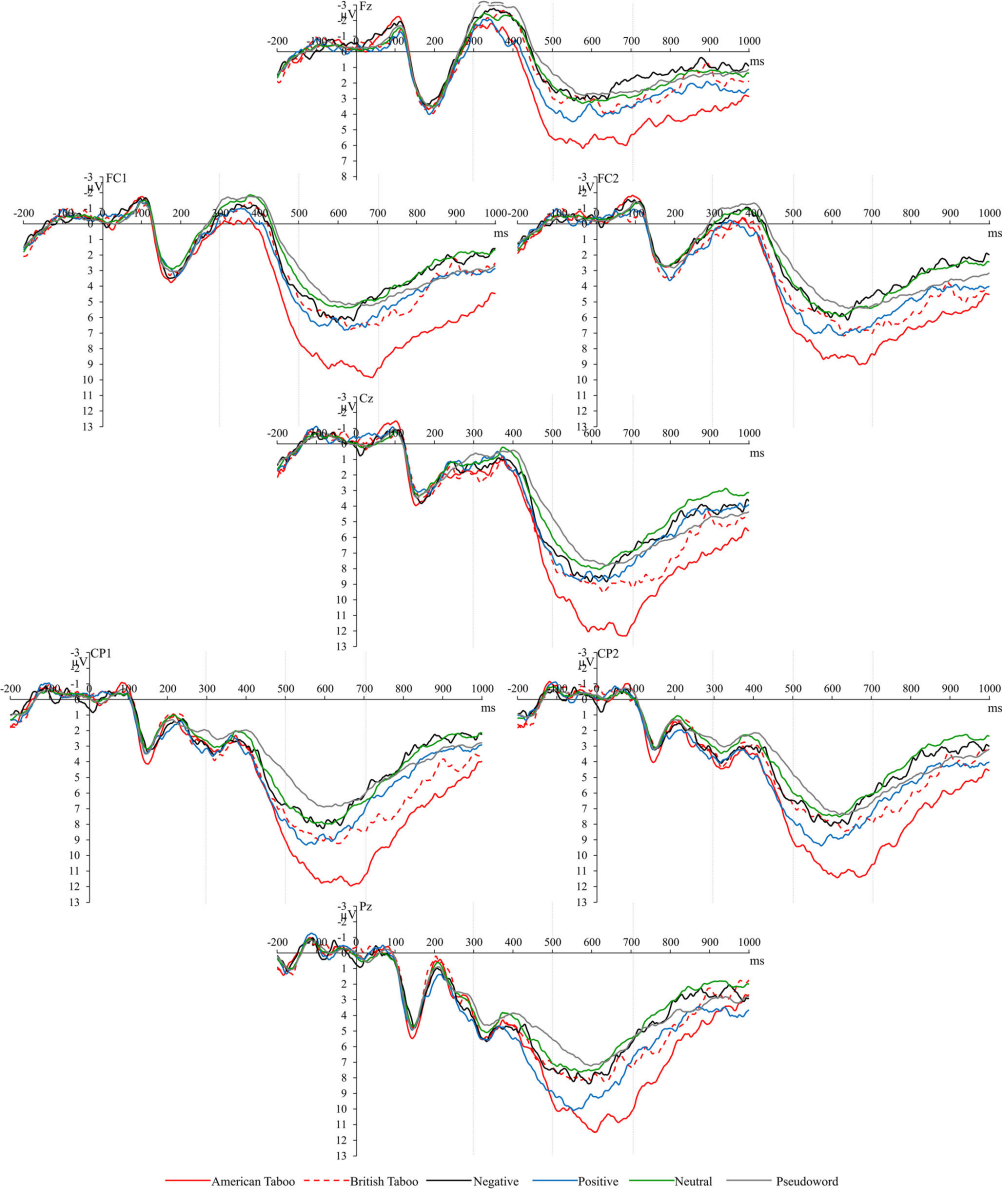
Grand average ERP waveforms showing the N400 and LPC for American taboo, British taboo, negative, positive, neutral, and pseudo-word conditions at all electrode locations (Fz, FC1, FC2, Cz, CP1, CP2, and Pz) included in the statistical analyses. Dashed vertical lines highlight the time segments used for the statistical analyses of N400 (300-500 ms) and LPC (500-700 ms)

Figure 4 illustrates the N400s for all conditions at all recording sites. Planned comparisons showed that pseudowords displayed significantly more negative amplitudes than all categories of words: pseudowords vs. AT: *F*(1,22) = 36.46, *p* < 0.001, *η*_*p*_^*2*^ = 0.624, pseudowords vs. BT: *F*(1,22) = 8.35, *p* = 0.009, *η*_*p*_^*2*^= .275, pseudowords vs. negative: *F*(1,22) = 5.31, *p* = 0.031, *η*_*p*_^*2*^ = 0.195, pseudowords vs. positive: *F*(1,22) = 16.66, *p* < 0.001, *η*_*p*_^*2*^ = 0.431, and pseudowords vs. neutral: *F*(1,22) = 4.71, *p* = 0.041, *η*_*p*_^*2*^ = 0.176.

#### LPC (500-700 ms)

**Fig. 5:**
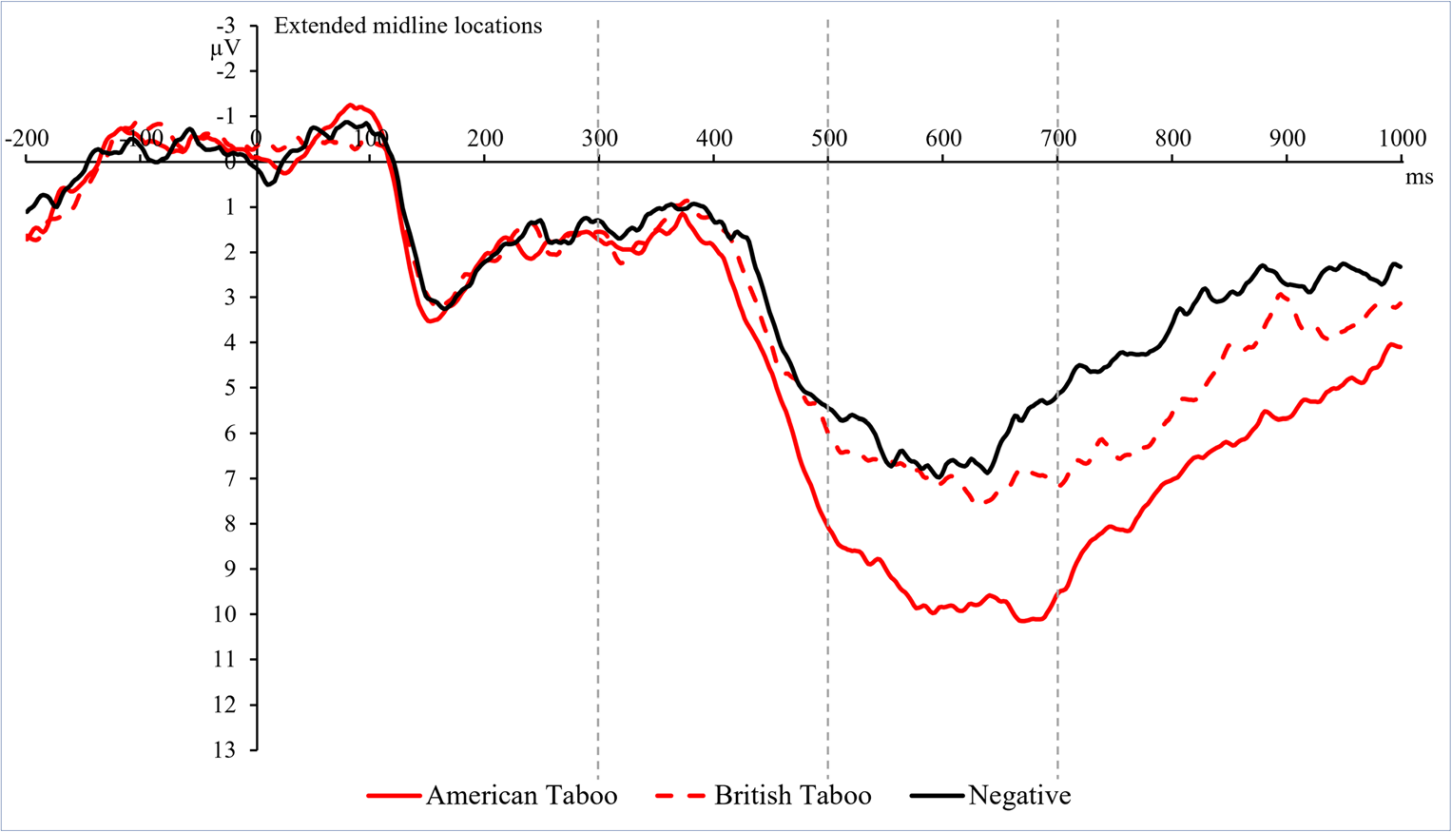
Grand average ERP waveforms highlighting waveforms for American taboo, British taboo, and negative words averaged across all electrode locations (Fz, FC1, FC2, Cz, CP1, CP2, and Pz) included in the statistical analyses. Dashed vertical lines indicate the time segments used for statistical analyses of N400 (300-500 ms) and LPC (500-700 ms)

**Fig. 6:**
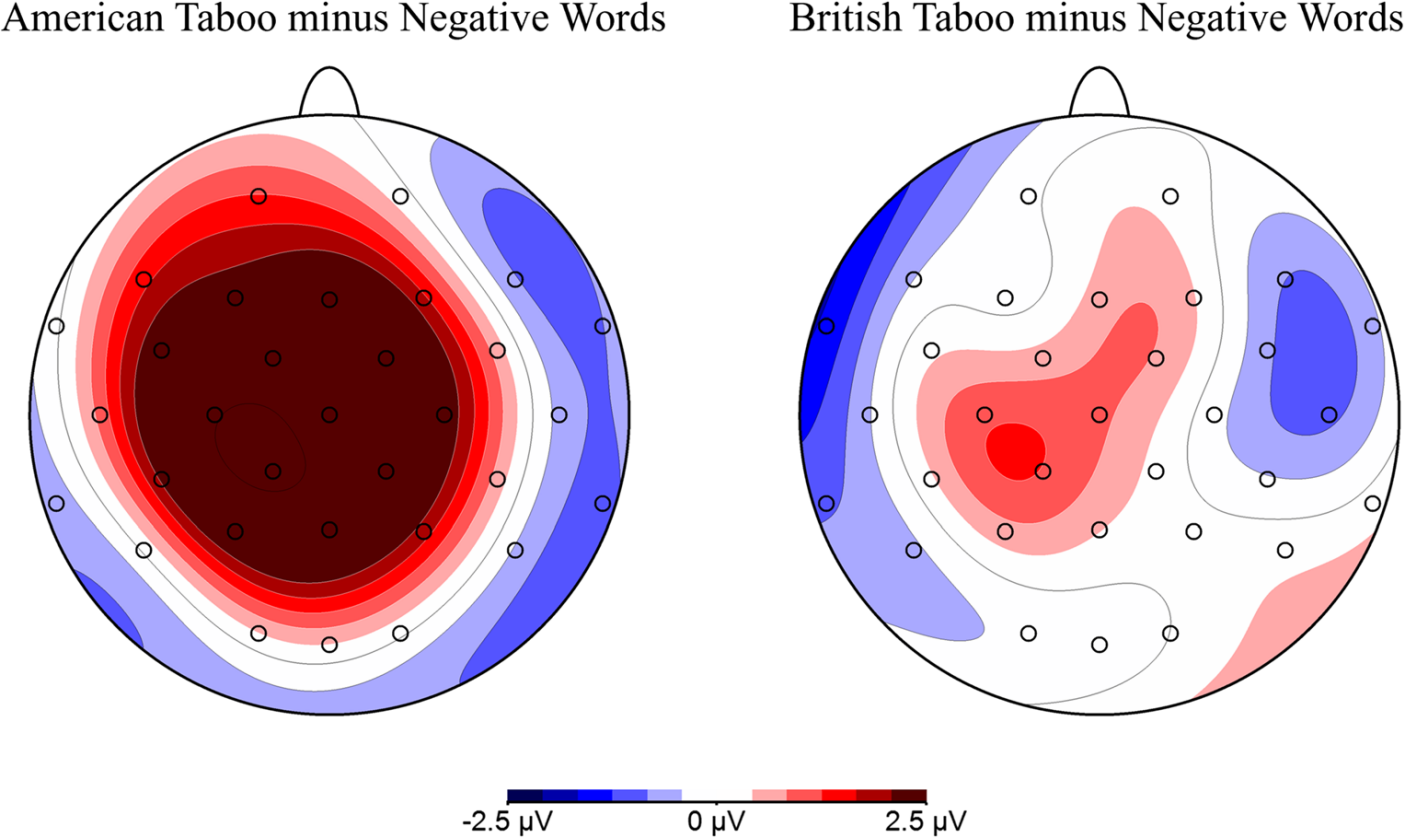
Voltage maps of ERP difference waves showing the LPC at 500-700 ms. The scalp distributions of the LPC (500-700 ms) effects were obtained by subtracting negative words from each of the American taboo (left) and British taboo (right) conditions. Spherical spline interpolation was used

Figure 4 shows the LPCs for all conditions at all recording sites. Figures 5 and 6 highlight the significantly larger LPC for AT than BT and negative words for illustration purposes. The repeated measures ANOVA yielded a main effect of word type, *F*(5,110) = 10.40, *p* < 0.001, *η*_*p*_^*2*^ = 0.321. AT elicited a larger positivity as compared to all other categories: AT vs. BT: *F*(1,22) = 31.31, *p* < 0.001, *η*_*p*_^*2*^ = 0.587, AT vs. negative: *F*(1,22) = 16.50, *p* = 0.002, *η*_*p*_^*2*^ = 0.429, AT vs. positive: *F*(1,22) = 7.71, *p* = 0.022, *η*_*p*_^*2*^ = 0.260, AT vs. neutral: *F*(1,22) = 32.59, *p* < 0.001, *η*_*p*_^*2*^ = 0.597, and AT vs. pseudowords: *F*(1,22) = 61.65, *p* < 0.001, *η*_*p*_^*2*^ = 0.737. BT showed an increased amplitude compared with pseudowords, *F*(1,22) = 6.22, *p* = 0.042, *η*_*p*_^*2*^ = 0.221. Positive words also showed an increased amplitude compared with pseudowords, *F*(1,22) = 9.80, *p* = 0.010, *η*_*p*_^*2*^= 0.308. No other significant differences were seen.

##### Exploratory correlation between item-wise LPC amplitudes and item (word) properties

**Fig. 7:**
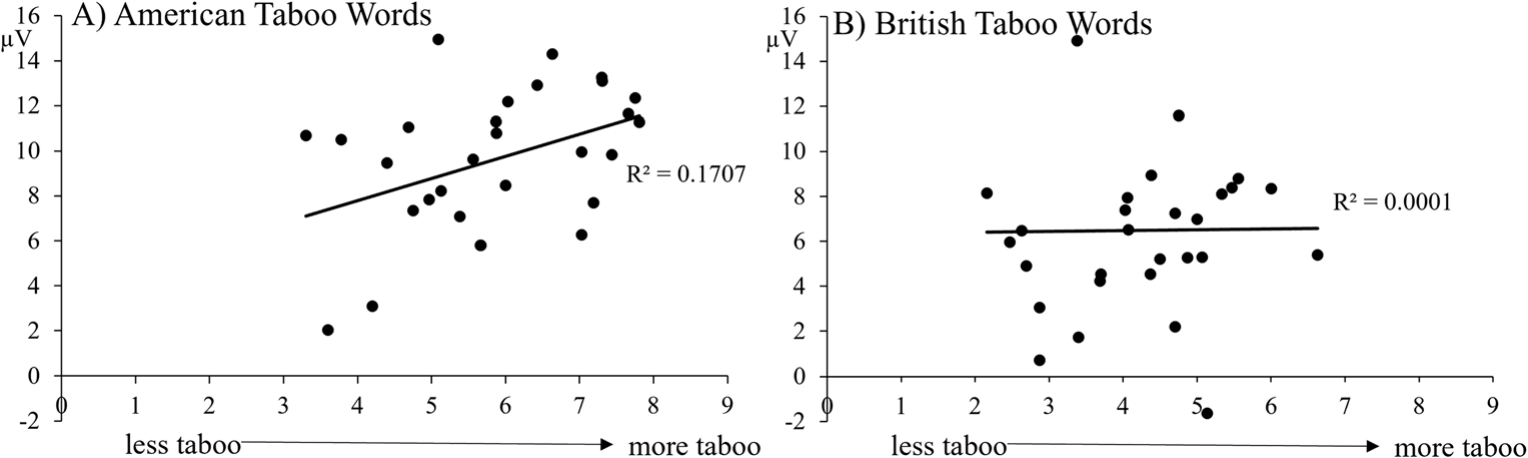
Scatter plots of correlations between tabooness ratings from the norming survey (x-axis) and mean amplitudes (y-axis) in the LPC time window (500-700 ms) for individual taboo words in the ERP experiment. Mean amplitudes were averaged across all electrodes included in the analyses (Fz, Cz, Pz, FC1, FC2, CP1, and CP2). (**A**) Significant positive correlation for American taboo words. (**B**) No significant correlation for British taboo words

We conducted an exploratory, stepwise regression analysis of valence, arousal, comprehension, and taboo ratings from the Norming Survey and the item-wise LPC amplitudes for each word to provide additional evidence that the differences in the LPC amplitudes can be attributed to the words’ tabooness. In this analysis, the mean LPC amplitudes were calculated for each AT and BT item and then correlated with each item’s rating scores. Please note that in this item-wise analysis data is compared across two independent samples of participants, the American group from the Norming Survey and the participants from the EEG experiment. This correlation represents a strong test of the contribution of tabooness to the LPC amplitude because it shows a relationship between data from two different samples. For AT, the regression returned taboo rating as a significant contributor to the LPC amplitudes, *r* = 0.41, *p* = 0.026, but none of the other variables were significant, *F*(1,27) = 5.6, *p* = 0.026, with an R^2^ of 0.413. For BT, the regression did not return a significant variable, *F*(1,27) = 0.1, *p* = 0.97, with an R^2^ of .02. Figure 7 illustrates this finding showing that for AT words (**A**) the LPC amplitude is more positive the higher the taboo rating, but no such effect was seen for BT (**B**).

## Discussion

The current study examined whether context of acquisition influences taboo word perception. We used taboo words due to their reliance on social context during acquisition. Additionally, we kept the test language constant to minimize the influences from proficiency and age of acquisition, by presenting American readers with taboo words in American and British English. We included nontaboo negative, positive, neutral, and pseudo-words for comparison. LDT behavioral results showed a processing advantage for American taboo words, which had a higher proportion of hits and faster RTs as compared to other categories. British taboo words had a lower proportion of hits and were slower than all other categories of words. ERP data showed that American taboo words elicited more positive LPC amplitudes as compared to British taboo and all other, nontaboo words. A regression analysis of item-wise LPC amplitudes and taboo ratings as well as comprehension, valence, and arousal showed that the more taboo an American taboo word was, the larger the LPC amplitude was. There was no difference in LPC amplitudes for British taboo words as compared to non-taboo words, nor was there a correlation between their taboo ratings and LPC amplitudes.

Results for the LPC support our hypothesis that taboo perception depends on their acquisition in social contexts. American taboo words had a higher proportion of hits (Figure 2) and more positive LPC amplitudes (Figures 5 and 6) than their negative, nontaboo counterparts, indicating that the two groups are distinct from one another. Given that the two categories were matched on all lexical characteristics measured except for tabooness and comprehension ratings (Table 2) and that the comprehension ratings were accounted for in the analyses, the difference in processing can be attributed to the taboo status of the words. This is in line with previous studies that compared negative and taboo words using ERPs (Donahoo and Lai 2020; Wabnitz et al. 2012). By contrast, British taboo words were also matched to negative words on all properties except for tabooness ratings (Table 2), but they did not differ from other categories in terms of LPC amplitudes (Figures 5 and 6). Thus, the results for American taboo words cannot be purely attributed to the explicit categorization of the words as taboo. A lack of social reinforcement (Vingerhoets et al. 2013), social threat (Wabnitz et al. 2012, 2016), and contextual memories (Pavlenko 2012) for British taboo words likely led to this difference in processing, as compared to their socially learned, American counterparts. Due to the American participants’ experience with American taboo words, whose use in certain contexts can have significant social impact (e.g., reprimands by authority figures, damaged relationships, social exclusion, etc.), these words show differences in processing compared to British taboo words that are specific to their experiential tabooness and cannot be attributed to taboo status alone. This is clear because the British taboo words, which American participants did recognize as taboo, but did not have the same immediate social experience with, do not show this taboo processing effects. Additionally, regression analysis showed that taboo ratings correlated with LPC amplitudes only for the socially-learned, American taboo words, while no correlation was present for British taboo words (Figure 7). This further supports our hypothesis, that the social nature of taboo requires taboo words to be learned in a social context for them to be processed as such, as well as more generally the *emotional context of learning* hypothesis (Caldwell-Harris 2014; Harris et al. 2006).

The N400 time window provides insight into the semantic processing of each word. We predicted that all categories of words would show less negative N400 amplitudes compared with pseudowords, indicating that they are recognized as words at the level of lexical semantics, consistent with previous literature (Kutas and Federmeier 2011). This was intended as a check to ensure that British taboo words were processed as words, given their lower comprehension scores. Results confirmed that lexical access was achieved for all categories of words, including British taboo words.

American taboos were recognized similarly fast as negative words when considering RTs in the lexical decision task, but slower than positive words. Insignificant differences in native taboo and negative word RTs are consistent with LDT studies conducted in both L1 and L2, wherein participants had to switch between languages (or dialects) during the LDT (Sulpizio et al. 2019). By contrast, Donahoo and Lai (2020) found taboo words had slower RTs compared with negative words. However, they conducted their study in only L1, where no form of code switching was required.

In behavioral results, British taboo words showed a significantly lower proportion of hits than all other categories, as well as slower RTs than American taboo words. The difference in RTs is likely related to the lower proportion of hits, with British taboo words being simply more difficult to recognize compared to words from the native dialect. This difference cannot be accounted for by frequency, which was matched across all categories. We suspect that this difference could stem from a difference in context of acquisition, dialectal status or a difference in word use frequency. Lack of contextual use may make it harder for these words to be accessed (Pavlenko 2012). Despite the differences in RT and hit proportion for British taboo words compared with American, these data do not sufficiently account for the absence of an enhanced LPC for British taboo compared with American taboo in ERPs. Additionally, only correct responses were used for the ERP analysis, thereby eliminating the possible effects of incorrectly processed words.

The behavioral results of the present study, compared with other studies of emotional and taboo words in L2, are mixed. Similar to negative words in L2 (Conrad et al. 2011; Chen et al. 2015), British taboo words were processed less accurately (lower proportion of hits) than negative, nontaboo words from L1 (Figure 2). However, our British Taboo words had fewer hits than all categories of words in the study. Furthermore, our results contrast with previous studies that showed no difference for taboo words in L2 compared with L1 in accuracy or RT (Eilola et al. 2007; Sulpizio et al. 2019). Our data showed significant delays for British taboo words (Figure 3) compared with American taboo words. It is possible that the results of previous taboo studies using L2 may have been influenced by participants’ L2 usage. Participants in those studies had considerably higher use of and exposure to their L2 than our participants had to British English.

Lastly, one might argue that these results may be driven by lower comprehension ratings, because British taboo words were rated significantly lower in how well they were understood than all other types of words in both the norming ratings and the post ERP ratings. We think that this is likely not the case, because first, despite the lower ratings, British taboo words were still understood, as the average ratings fell between 3 (generally understand) and 4 (understand well). Second, the difference in N400 amplitude compared with pseudowords also was significant for British taboo words, showing that they were comprehended as meaningful words with lexical access occurring (Kutas and Federmeier 2011). Third, only correct trials, correctly recognized and comprehended words, were included in the ERP analysis. These differences in behavioral outcomes for British taboo words then are most likely due to the difficultly of LDT with nonnative words, rather than taboo, emotional, or comprehension effects.

The positive words elicited larger LPCs than negative and neutral words. Previous research has shown that positive words do not differ from negative in LPC amplitudes, with both having higher amplitudes than neutral in a LDT paradigm (Citron 2012). However, increased amplitudes for positive words are not unheard of (Kissler et al. 2009). The positive words were included in the task set to ensure that we were thorough in our investigation and included the full range of the valence scale, as well as to connect with previous studies of emotional word processing in L1 and L2 (Chen et al. 2015; Conrad et al. 2011; Opitz and Degner 2012; Sulpizio et al. 2019). This discrepancy may result from a difference in design: Our study included two kinds of taboo words, as well as negative, neutral, positive, and pseudo words. As a task set, American taboos might have been equated with negative words in our participants’ minds, causing the positive words to become an infrequent deviant that stood out from the more frequent items (taboo/negative or neutral). In other words, we might have inadvertently created an emotional oddball paradigm (Strange et al. 2000), showing a facilitation of the deviant, positive words. The difference in LPC between positive and both, negative and neutral words may be the result of a P300 effect for the positive words caused by this oddball paradigm (Schluter and Bermeitinger 2017).

One meaningful limitation of our study is that our American English speaking participants’ comprehension ratings of British taboo words were significantly lower than of American taboo words. This may present a confound, given that proficiency has been shown to influence emotional processing and offensiveness in L2 (Eilola et al. 2007; Wang 2019). To determine if the reduced comprehension could account for the LPC difference between British and American taboo words, we conducted an ERP analysis using only highly comprehended words (those rated “generally understand,” “understand well,” or “understand completely”). These data showed a similar pattern of LPC results as compared to the currently reported, less well comprehended set, but were not reported due to a low trial count (~10 trials) per condition. Additionally, previous studies have shown that, whereas social use of taboo words is positively correlated with understanding, it does not influence offensiveness judgements for taboo words in non-native dialects of L1 (Dewaele 2015). We therefore think it unlikely that the current LPC results for American and British taboo words are solely due to differences in comprehension between American and British taboos. To amend this, the current study could be repeated using native speakers of British English, who indicated no difference in comprehension between dialects (Table 1; Dewaele 2015), or native speakers of American English with greater contact with British English. This would reflect methods used in previous bilingual studies of emotion (Conrad et al. 2011; Opitz and Degner 2012).

Although this study utilized dialect rather than distinct languages, our results support findings that have previously been found in bilingual studies. Bilinguals show the influence of social context on their reported perception of language, having reported their preference for taboo words from their naturalistically-acquired L1 as opposed to an academically acquired L2 (Dewaele 2010, 2011). However, they may find L2 taboo words equally cathartic when L2 is learned in a naturalistic context (Dewaele 2017). Additionally, our results are consistent with pervious physiological studies where bilinguals had a greater response to taboo words in the language that they learned in a social context (Eilola and Havelka 2011; Harris 2004; Harris et al. 2003). While our experiment was confined to a single language for the purpose of controlling proficiency, the effects of social context of acquisition extend to the bilingual domain.

Our findings align with the *emotional context of learning* hypothesis of language acquisition (Caldwell-Harris 2014; Harris et al. 2006), emphasizing that how a (taboo) word is learned influences how it is processed. Similar to emotional words, taboo words require learning and use in a social context, in other words experiencing the taboo to acquire taboo effects. Our participants had little direct contact with speakers of British English, preventing the cultural and social factors that make up naturalistic language acquisition (Labov 1987). This prevented or impeded participants from processing the additional social aspect of nonnative taboo words (Donahoo and Lai 2020; Reilly et al. 2020). Our results furthermore align with reports from highly proficient bilinguals that swearing is less cathartic in L2 (Dewaele 2010, 2011), because recognizing or being aware of the taboo meaning of non-native words is not enough to replace their deeply integrated social threat that comes when they are used in interaction. While nonnative taboo words retained their taboo designation in our survey data, they did not show the corresponding instance of taboo processing in ERPs, demonstrating that ERP recordings can illustrate subtle differences that behavioral data alone cannot.

Our data strongly suggest that acquisition in a social context wherein the taboo is present is required for the taboo meaning of the word to be learned. This has previously been suggested by reports of equal swearing in both languages by simultaneous bilinguals who learned both languages in social context (Wickham 2019). This corresponds to our ERP results seen in the native taboo words, where their processing was correlated to their socially learned taboo and was not connected to their comprehension. In sum, our results show that social interaction, rather than proficiency, influences taboo perception.

The current study isolated the contribution of social interaction to taboo word processing by utilizing taboo words from participants’ native (American) and nonnative (British) dialects. While the non-native taboo words were understood explicitly as taboo, their social meaning was not processed. This indicates that taboo words encountered in naturalistic contexts elicit richer emotional and social responses at the neural level compared to taboo words that did not have such a context during acquisition. Our results demonstrate that acquiring language through social interaction influences the affective meaning of language.

### Acknowledgments and Funding information

This research was supported by a College of Wooster APEX Fellowship grant, which was made possible by the Wilson APEX Fellowship Endowment, by the Henry J. Copeland Independent Study Fund grant and the Sophomore Research program at The College of Wooster. The authors thank Olivia Ogle for help with data coding and Stanley Donahoo for assisting in stimuli creation and planning.

## Figures and Tables

**Figure:**
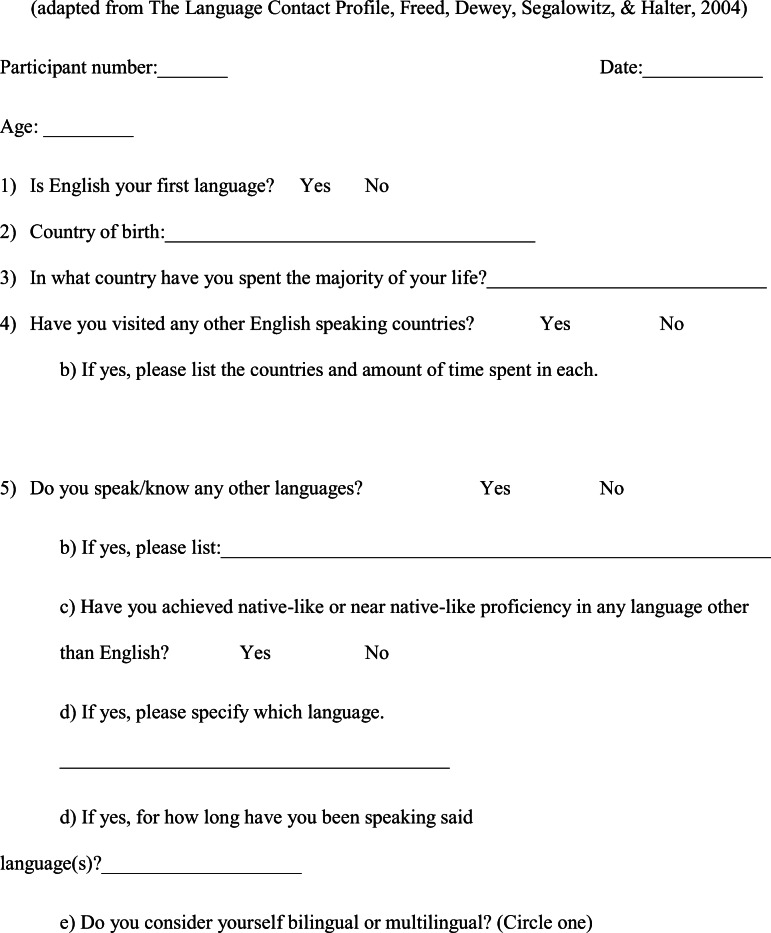

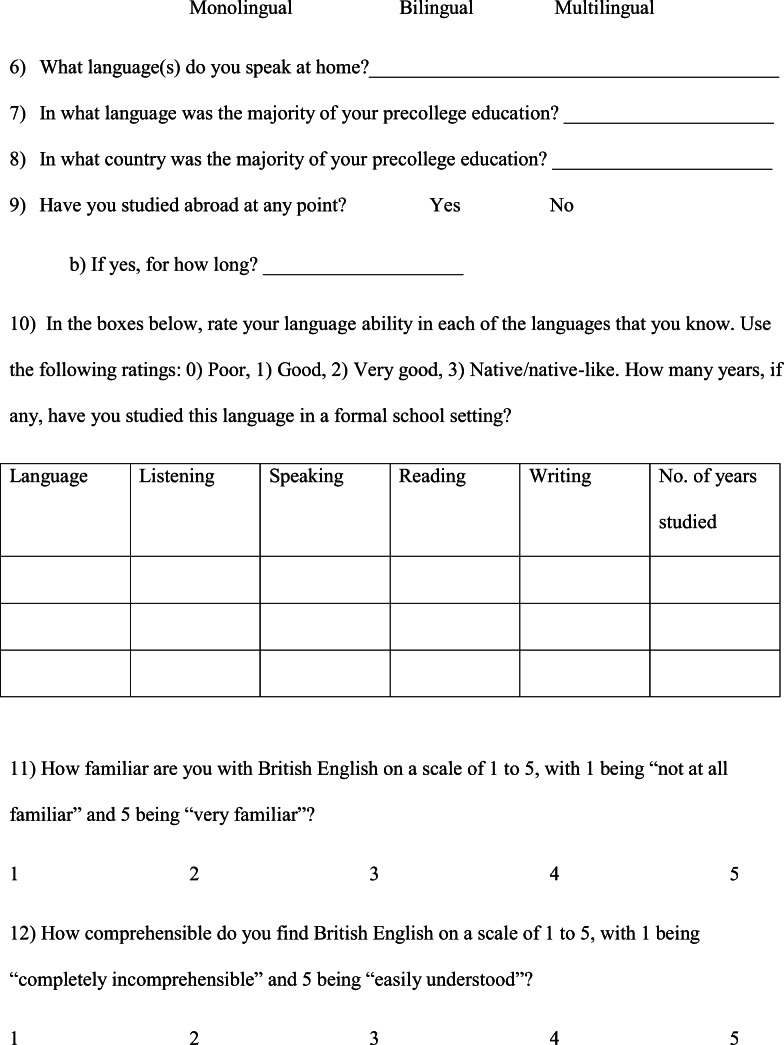

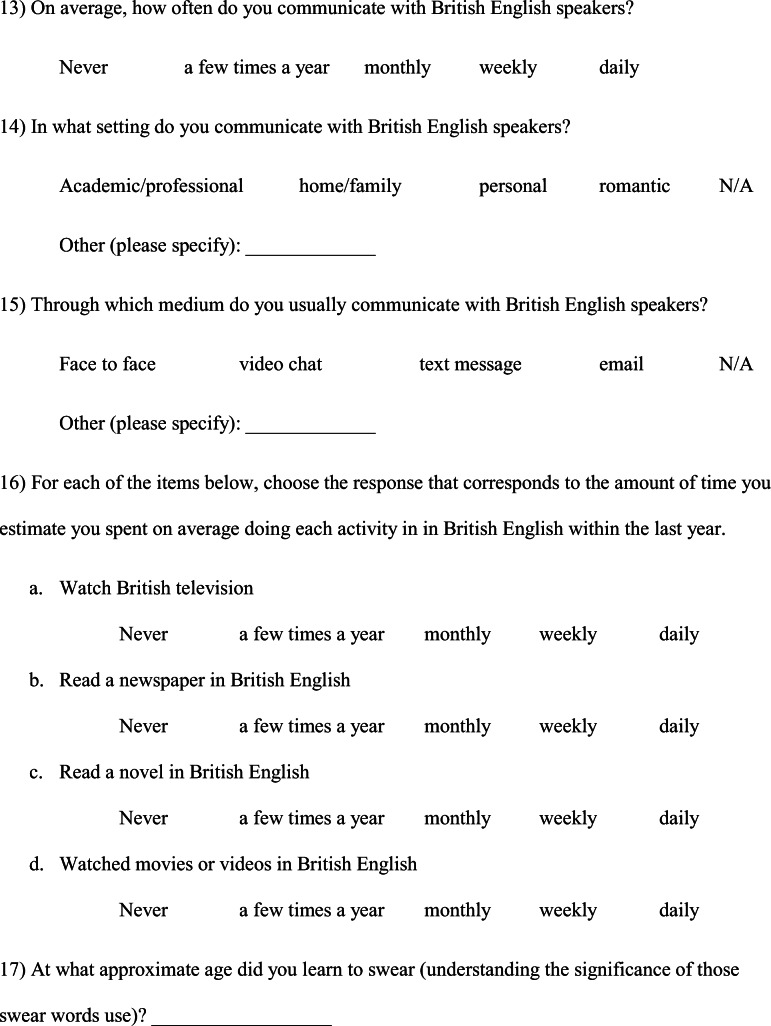

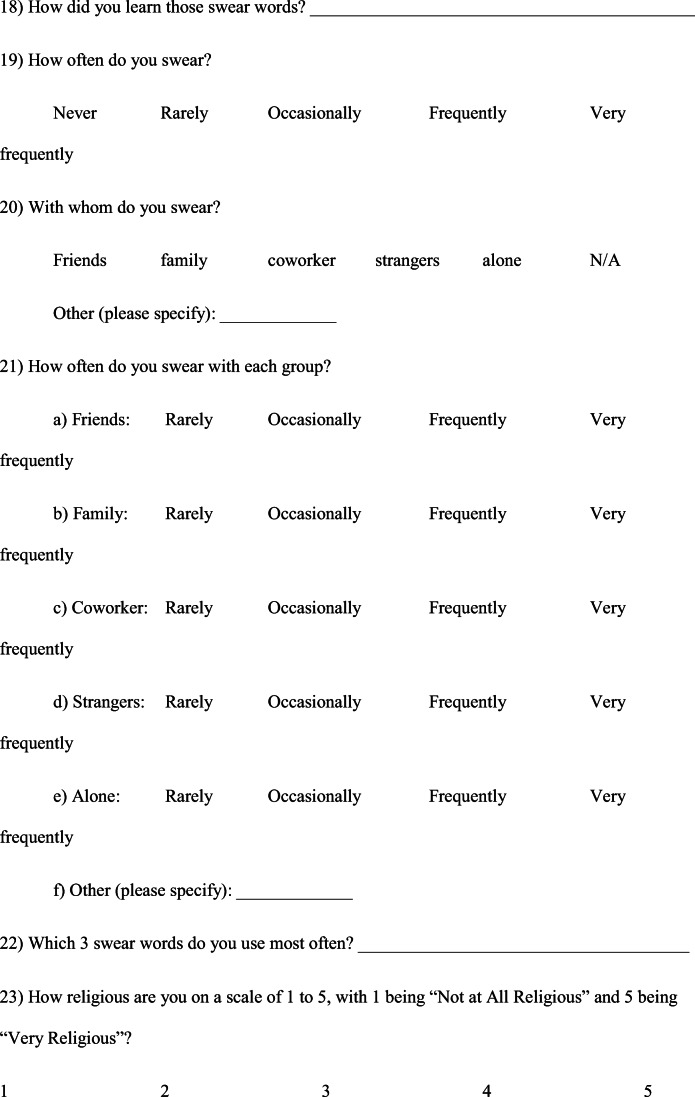
Background Questionnaire

## Appendix A

**Appexdix B:** Demographic data collected from adapted Language Contact Profile (Appendix A) for nonexcluded participants

	Percentage (%)		
English L1	100		
Born in United States	95	Other country of birth:	
		China (1)	
Majority of life in United States	100		
Visited other English-speaking countries	70	Countries visited:	Average time spent (days):
		Canada (6)	26
		England (6)	12
		Australia (1)	90
Self-declared bilingual	20	Second languages:	Average years learned:
		French (2)	13
		German (1)	20
		Spanish (1)	14
Self-declared multilingual	5	Number of additional languages:	Fluent languages:
		2 (1)	Spanish, Portuguese
	Likert Rating (1-5)		
Familiarity (British)	3.1		
Comprehension (British)	3.7		
Religiousity	1.75		
	Frequency rating, 1-never to 5-daily		
Personal contact (British)	1.9		
Media contact (British)	Type of media:		
	TV	2.1	
	News	1.4	
	Novels	1.5	
	Magazines	2.3	
	Frequency rating, 1-rarely to 4-very frequently		
Frequency of swearing	3.5		

**Appendix C:** Stimuli by word category (non-excluded)

American taboo	British taboo	Negative	Positive	Neutral			
boner	arse	puck	pup	curd	totem	sassy	swoop
chink	blithering	clang	bouncy	maroon	hoist	madden	whiskey
coon	bloody	wrinkle	glimmer	nip	rouse	bionic	curling
cocksucker	bollocks	mite	fillet	stair	coven	flares	jug
douche	bugger	burner	boating	mingle	slurp	rodeo	boxer
dyke	bum	charred	peach	curling	watcher	gullet	whistle
fag	dodgy	stuffy	juicy	manned	crater	unborn	limber
faggot	git	beg	wink	tango	bleep	tempt	consoled
fart	harlot	shiver	glee	spur	kicker	bleach	stove
fucker	lout	avenge	bubbly	clout	rapture	medal	hawk
goddamn	ninny	crank	doggy	rumble	fiver	shocking	rattle
hoe	prick	bash	charmer	stag	cunning	blaze	sheltered
jackass	shag	boa	sundae	camper	hatch	lair	coarse
loser	slag	fumble	zing	hose	barman	prank	knot
moron	tart	barbed	lavish	swell	burp	waxed	reverent
queer	trollop	clatter	fudge	dub	ledge	racy	stool
redskin	twat	revolver	hoot	tout	sniff	buzzer	lawn
retard	twit	moody	buffet	dibs	steep	boil	vest
taint	wank	hoax	pastry	greased	trooper	bikini	bandage
skank	wanker	thud	twirl	hunch	gobble	defy	errand
kyke	sodding	horde	chic	stash	rum	jumpy	cork
douchebag	knockers	feud	soar	brawn	bingo	sneak	golfer
buttfuck	arsehat	vanish	clap	ravine	crave	strut	neurotic
clit	gobshite	jab	woof	soot	wiggle	groin	diver
cum	knobhead	sour	spunk	lasso	raffle	stun	garter
dildo	nutter	flee	feast	clink	bonkers	bearded	lavish
kink	poxy	sling	tasty	gooey	booze	bees	tidy
sucker	shite	parting	giddy	reap	livery	gush	pancakes
schmuck	tosser	chore	shimmer	kilt	flicker	howl	aloof
masturbate	cheeky	snag	witty	bump	loot	gasp	icebox

**Appendix D:** Comparison of words to pseudowords

	AT	BT	Negative	Positive	Neutral
	t-value(df)	t-value(df)	t-value(df)	t-value(df)	t-value(df)
Accuracy	3.26 (22)**	1.76 (22)	1.47 (22)	4.79 (22)***	2.3 (22)*
RT (ms)	7.02 (22)***	2.65 (22)*	6.07 (22)***	10.03 (22)***	8.84 (22)***

*Significant differences (alpha < 0.05).

**Significant differences (alpha < 0.01).

***Significant differences (alpha < .0001).
